# The Nature and Treatment of Insanity
*Remarks on Insanity, its Nature and Treatment. By Henry Munro, M.B., Oxon., Fellow of the Royal College of Physicians. Churchill: London. (Second Notice.)


**Published:** 1851-07-01

**Authors:** 


### ART. III.—THE NATURE AND TREATMENT OF INSANITY.*

WE concluded our former notice of the first part of Dr. Munro's work on Insanity by quoting a graphic description of the mental state in old age, illustrated by a beautiful metaphor, which, although used in a somewhat similar sense by Pliny, and gracefully employed by Sir Humphrey Davy in his "Salmonia," to typify the various characteristics of childhood, manhood, and old age, has lost none of its charms in passing through the cultivated mind of our author. Indeed, candour compels us to state, that much of the interest of the work is owing to the eloquent style of the writer; and its usefulness will be found, rather in the ethical reflections which have been deduced from well-known facts, than in any originality of thought as to the "nature" of insanity, or from any novel plan in its "treatment." There have not been wanting those who have argued, that there is something in the collegiate halls, and "academic bowers" of Oxford calculated to inspire the mind with holy and elevated thoughts; be this as it may, our author does honour to his Alma Mater, both by the purity of his sentiments, and the graceful elegances of his style. With the philosophy and spirit of the following remarks we cordially agree:—

"Above all, the sense of this principle within us points with the finger of unerring truth to that great and universal condition stamped upon our nature at the fall of man, namely, that we are born for labour; that while work is our doom, it may become our chief blessing, as long as we attempt not to resist its claims upon us. There are some whom we may call the first-born of the creation, who seem to have learnt this lesson instinctively; they have a fire within them which will find fuel for its expenditure; and while they enjoy health they do most assuredly find such fuel wherever they may be thrown; work to them is not tedious labour, but the absence of it is; to these ethereal and practical beings the rest of the world succumbs in the end, whatever resistance may be shown by zealous and little minds; and

* Remarks on Insanity, its Nature and Treatment. By Henry Munro, M.B., Oxon., Fellow of the Royal College of Physicians. Churchill: London. (SECOND NOTICE.)

when such spirits are enlightened with a ray from heaven, the passage of their life seems like a transit of a beautiful dream; when they are taken away from us by the hand of death, their blessing ceases not, but casts a radiance on all those who have valued them while with them. Such beings are, however, like the single planets among the stars—few, and rarely loved by him who gazes on the depressing gloom around him; and, what is more than this, while they shine thus for others, they rejoice in the glow of their own light, as they know the source from whence it springs. People of the excitable temperament peculiarly require a full realization of this great truth, namely, that thought, to be healthy, must culminate in acts; for if they find not a field suited to their energies, that which might have made them surpass their fellows (namely, an ardent spirit,) by re-acting on themselves, causes them to fall behind their fellows, since they cannot maintain the virtue even of a more phlegmatic disposition, namely, a serene tenor of mind; and thus they become not only a burden to themselves, but a burden to other people. A state of depression, however, they might still escape, if they would, while they have the power, employ their faculties. The force of this great truth is shown most strikingly in the simplest forms of human nature, namely, the phenomena of infancy; little children are in constant action, every thought drives them to act, and they are happy. The higher and more complex nature of grown man, whose first business it is, not to be the slave of the dictates of his animal nature, but to be their master, and to raise that nature to a world above him (whose influence he feels, though he cannot see,) cannot enjoy this simplicity of nervous action; and therefore when he energizes in abstract, intellectual acts, he suffers many ailments which the simpler state of childhood is free from. This is, however, his destiny, and one not to be resisted, but only kept within certain bounds; for while it is, or ought to be, the peculiar work of childhood (or rather of those who have its guidance) to arrive at the perfect maturity of the grown man, it is the work of man to stand with his fellows between the Creator and the created: and while, in the image of his Maker, he draws his strength from above, he should let its blessed influence distil upon the lower world around him, over which he rules, but the dominion over which he holds, only that it may be made tributary to its great Creator's honour. Therefore the work of man in health is not simply to seek the full enjoyment of his animal nature, but to take care to keep that existence in such a condition that it is suited to be a fit channel of its higher nature. This, however, is not the condition of the insane, and he should not be looked upon in this light; for he has fallen, through disease and sorrow, from his high estate; he is, as it were, again in the position of the child, and it should be the object of every philanthropic mind to replace him as far as may be in that noble position."

The work is rich in elevated thoughts, clothed in the same poetic language as the above; and although we have long been made familiar with the same idea—namely, the dignity of labour—by the writings of

Chalmers, Channing, Carlyle, Melville, and others, still it is refreshing to meet with such beautiful episodes in a work of medicine, especially when they seem to spring, as in the above instance, from the subject, and are introduced for a practical purpose. Perhaps we ought to state that the author's sensitive and accomplished mind has induced him to indulge more extensively than many would approve, upon the ethical branch of this interesting science; more especially as the work mainly professes to discuss the pathological condition upon which insanity is dependent, and the appropriate treatment for its removal and cure. He delights in every opportunity of indulging those feelings which the phrenologists allocate in the organs of "ideality" and "veneration." Whole pages are occupied by graceful descriptions of the emotions which spring up in the mind "in solitude," "in dreaming," and "in the dim hour of cock-crowing," "when the shadowy forms around us become the creatures of our own fancy," or "when the first silver cloud floats along in its glowing track, giving colour and more palpable existence to the very air itself, and shining in the horizon as the herald of hope;" and in those deep umbrageous woods whither the poet and the lover flee from the busy scenes of life to enjoy "sensations of a nature more exquisite than any diversified excitement could ensure." On these scenes and moments the author expatiates with all the exuberance of a poet. In his pages we meet with the glowing warmth of Zimmerman and the deep pathos of Petrarch, blended with a portion of the mystic speculations of Coleridge and Keats; and, may we not add, a slight tincture of that sentimental and sensational philosophy, which, originating in the mind of Froude, has awoke the lyre of Keble, and spread its fascination and power over some of the most earnest minds of the present generation? This large ideality has, in many parts of the work, been productive of beautiful speculations, but it has also, in some instances, prevented the author from taking an accurate view of facts as they really are, and has so influenced and moulded his theories, that, to use his own poetic imagery, they "remind us, both by their beauty and fragile existence, of the scenes and associations offered to the senses when we stand in some dim Gothic building, and gaze through the coloured windows upon the scene of life and light without. The daylight, as seen through the red and yellow lights, and all the fantastic furniture of windows, appears ever a glowing sunset: dreams of beauty and fancy are raised, which vanish instantly when the full light is revealed on leaving those solemn shades; and the least rough handling of those intenser thoughts, like the wind which rattles the crystal casement, remind us instantly how frail and delicate our scenes of beauty are." To some of these metaphysical speculations we shall hereafter refer.

In our former notice, we demurred to the general applicability of Dr. Munro's theory of the cause of insanity; and further reflection confirms the view we at that time espoused. Every day's experience reveals to us a large majority of cases in whom there is "excitement without power;" but these cases are as frequent in other maladies as in insanity; and this latter affection is no more dependent upon loss of nervous tone, the consequence of deficient vital power, than is pneumonia or gout. We regard the "theory" as a revival, for a special pathology, of the exploded hypothesis of Brown, and cannot, therefore, award to it the high praise of originality. Indeed, the author himself states, that his "proposition escapes both the honours and anxieties of originality;" although, in other portions of the work, he speaks of it as "my theory," and seems to regard himself as enunciating new truths upon a most momentous subject. Spurzheim, in 1817, writes—

"The idiopathic causes of insanity, in its most extensive signification, either exist from birth, or originate from later events. These latter are mechanical,—that is, the effect of a violent cause; or dynamical, the result of the deranged functions of vitality, viz., of the vital powers."*

So far the theory must be true, inasmuch as the same phraseology would apply to every disease. If *inflammation* be invariably the result of depressed vital power, then, indeed, we grant the truth of Dr. Henry Munro's theory, as embracing a platitude, or a truism, from which no one can dissent, but which, however, leaves the malady as inexplicable as before he penned a single line of his beautiful essay. It places us before the unsolved question of, What is the vital power? What, indeed, is the brain itself? To use the language of one with whose writings our author is evidently familiar, what do we mean, what do we perceive, in the eye itself, or in the flesh itself?

"Carbon and nitrogen, oxygen and hydrogen, sulphur, phosphorus, and one or two metals and metallic bases, constitute the whole. It cannot be these, therefore, that we mean by an eye, by our body. But perhaps it may be a particular combination of these. But here comes a question: *In this term, do you, or do you not, include the principle, the operating cause of the combination?* If not, then detach this eye from the body. Look steadily at it, as it might lie on the marble slab of a dissecting-room. Say it were the eye of a murderer, a Bellingham; or the eye of a murdered patriot, a Sidney! Behold it—handle it, with its various accompaniments or constituent parts, of tendon, ligament, membrane, blood-vessel, gland, humours; its nerves of sense, of sensation, and of motion. Alas! all these names, like that of the organ itself, are so many anachronisms, figures of speech to express that which has been—as when the guide points, with his finger, to a heap of stones, and tells the traveller, 'That is Babylon, or Persepolis.' Is this cold

* Observations on Insanity, p. 120.

jelly 'the light of the body?' Is this the *micranthropos* in the marvellous microcosm? Is this what you mean when you well define the eye as the telescope and mirror of the soul, the seat and agent of an almost magical power? Pursue the same inquisition with every other part of the body, whether integral or simply ingredient, and let a Berzelius or a Hatchett be your interpreter, and demonstrate to you what it is that in each actually meets your senses. . . . . . . . . . It is to the coarseness of our senses, or rather to the defect and limitation of our percipient faculty, that the visible object appears the same even for a moment. The characters which I am now shaping on this paper abide. Not only the forms remain the same, but the particles of the colouring stuff are fixed, and, for an indefinite period at least, remain the same. But the particles that constitute the size, the visibility, of an *organic* structure, are in perpetual flux. They are to the combining and constitutive power, as the pulses of air to the voice of a discourser, or of one who sings a roundelay. . . . . But perhaps the material particles possess this combining power by inherent reciprocal attractions, repulsions, and elective affinities, and are themselves the joint artists of their own combinations? I will not reply, though well I might, that this would be to solve one problem by another, and merely to shift the mystery. It will be sufficient to remind the thoughtful querist, that even herein consists the essential difference, the contradistinction of an organ from a machine; that not only the characteristic shape is evolved from the invisible central power, but the material mass itself is acquired by assimilation. The germinal power of the plant transmutes the fixed air and the elementary base of water into grass or leaves; and on these the organific principle in the ox or the elephant exercises an alchemy still more stupendous. As the unseen agency weaves its magic eddies, the foliage becomes indifferently the bone and its marrow, the pulpy brain, or the solid ivory. That what you see is blood, is flesh, is itself the work, or shall I say the translucence of the invisible energy, which soon surrenders, or abandons them to inferior powers (for there is no power or charm in the activities of nature), which repeat a similar metamorphosis according to their kind; these are not fancies, conjectures, or even hypotheses, but facts; *to deny which is impossible*, not to reflect on which is ignominious."*

If, therefore, by the term "loss of vital power," is meant a disturbance, or suspension of that "unseen agency" which presides over nutrition, and constitutes, in its regular action, *health*, we grant the premises, and follow the conclusions of the work before us; not, however, as proclaiming anything new, but as simply reiterating a truth, which has been tacitly admitted by all, but which, from its elementary character, has been left to be "understood," (to use a phrase of early days) by most writers on pathology. Dr. Hitchman, whose investigations have been referred to by Dr. H. Munro at page 97 of his work, states—

* Coleridge.

"I believe that before the scalpel can reveal opacity, thickening, and infiltration of the membranes, or congestion, inflammation, softness or hardness of the medullary matter, *there must have been great and important changes going on*, and that necroscopic appearances ought to be regarded more as results than causes—*as the effects* rather *than the source* of the malady whose nature and habitation we are anxious to explore."—*Lancet*, Nov. 27, 1847.

In our own pages, where the pathology of insanity has recently been largely entered upon by the same writer, its causes have been tabulated as arising—primarily, from some change in the brain, induced by psychical causes, such as grief, joy, prolonged intellectual exertion, &c.; and also by physical or somatic changes, such as impaired nutrition, irregular development, and mechanical violence;—and, secondarily, by blood diseases, arrest of excretions, sympathy with remote organs, &c. In Dr. Henry Munro's volume the malady is ascribed "to loss of nervous power, consequent on loss of vitality," and this "loss of vitality" is produced in three distinct modes:—

"First, external poisons entering and poisoning the system. Second, moral shocks *reacting* upon the nervous system, and exhausting or rather depressing its vigour. Thirdly, constitutional and internal sources of nervous, and vital depression—some of them healthy and consequent on natural depression, such as that of sleep and dreaming —some of them consequent on the disease of internal organs, such as that condition when urea or any animal poison or excretion is thrown back upon the system—and a third species, which belongs to this class of vital depression, namely, an *unhealthy* state of depressed vitality, consequent upon an *unnatural* exhaustibility of the special organ, the sensorium, but not dependent essentially upon disease of any other organ."—p. 74.

The table of each of these writers embraces the same causes differently arranged; but while the former avows that "each and all of these causes act by producing *functional disturbance* or *structural change* in the vesicular neurine (grey matter) of the encephalon, and thus produce insanity," the latter reaches apparently higher in causation, by ascribing all the wondrous phenomena exhibited in this disease to "loss of vitality." We do not wholly agree with either of these pathologists, between whom there is, perhaps, in reality, no great difference of opinion, for Dr. Hitchman distinctly states, that there are links of causation beyond the one that he has described—beyond, indeed, the reach of human investigation. "Although there *must be* some one *elementary* change, upon which insanity is dependent, it is doubtful whether it will ever be demonstrated: there will occur a multiplicity of visible changes in that structure (supra), by any of which insanity may be caused through embracing the elementary one; and these alone will be demonstrated by future research,"—and he seems to point out the *vital* endowments of organs as a divine fact to be reverentially admitted; as a dynamic principle, the precise *nature* of which is beyond the grasp of human philosophy, but which presides over and controls all the manifestations of health and disease. This theory is more definite and precise than that of a local "loss of vital power," and is more within the reach of positive induction. It is capable of being substantiated or disproved by well-observed facts; and whilst it also admits of being upheld as true, by appealing to the results of medical treatment, it does not depend for its support upon so illusory a basis as the reputed effects of any therapeutical agent. If insanity be the result of a loss of vital power, what are the indications of an excess of the vital force? Many cases are met with in practice in whom the local loss of blood relieves the malady; in whom nauseating doses of tartarized antimony are productive of more benefit than exercise or diet, although we admit that they are more rare than in those days when the patients at Bethlem Hospital were regularly bled—or when Dr. Rush resorted to the heroic practice of taking as many as 470 ounces of blood from one patient. Let us not be misunderstood—we *know* that, *in the great majority of instances, insanity is an asthenic malady,* requiring tonic treatment, and we rejoice to agree with the talented writer in his many judicious remarks upon this head. This fact is, however, as old as the days of Pinel—was strenuously contended for by Mr. Nesse Hill in 1817—has been advocated by Dr. Hitchman and others, in their respective lectures and works—has been invariably upheld by the writers in this Journal, and forms, perhaps, one of the most perfect specimens of unity of opinion to be found in the whole range of medical literature—inasmuch as, out of fifty-one superintendents of asylums whose experience was called for by the Commissioners in Lunacy in 1846, no less than forty-eight recommended a generous diet, and abstinence from general depletion. Still there remain cases of "sthenic insanity," in whom there can be detected no more traces of loss of vital power, than are to be found in any case of acute peritonitis, of rheumatism, or pneumonia, in whom, in order to cure quickly and safely, we must resort to antiphlogistic measures, or our patient will rapidly sink into perfect fatuity, or glide to that "bourne from whence no traveller returns." We repeat again our deliberate conviction, that no fixed rule can be given for *every* case, but the judicious physician will treat each one according to its respective acquirements, regardless alike of the theories or the men which may happen to be popular at the time; knowing full well that the cure of the patient is the praise of the physician, and that there is no award so precious as the consciousness of having thought as much and acted as well as our judgment, experience, and the well attested observation of honest minds, unbiassed by modern theories, enabled us to do.

Dr. Munro argues, that the presence of "*inflammatory action in the brain would corroborate the theory that insanity arises from nervous and vital depression.*" This, if understood in the sense we have before indicated, involves an axiom, which we have no desire to dispute; but as we have also added, it equally applies to those acute diseases for which active *depletion* has hitherto been found necessary and advantageous, and throws no additional light upon the obscurities of cerebral disease.

"2nd. *The best argument that inflammation of the brain is no essential condition of insanity is, that extravagant insanity so often exists without the least trace of it.*" We have never regarded inflammation as an *essential* condition of insanity—but maintain that it is sometimes *a condition,* and demur to the position that it *often* exists without the *least* trace of it. If the brain in *acute* cases of mania were examined *immediately* after death, and prior to the head being placed in a dependent or erect condition; in other words, if the head were kept in a horizontal position, these traces would be more frequently observed than they now are—not, indeed, in the general structure of the brain—but as evidenced in a congested condition of the pia mater, and a roseate hue of some parts, or the whole of the convolutions; even, however, if these appearances should not be detected after death, it would be no proof that they did not exist prior to dissolution; we have only to observe the effects of syncope upon an inflamed conjunctiva, to comprehend the changes which may ensue at death, in so delicate a structure as the vesicular neurine of the cerebrum. In his third argument, Dr. Henry Munro urges, "*that inflammation is not a source of disease at all, but rather a consequence;*" this must be granted, if we argue up to first causes, but then "*depressed vital power*" leaves us in the same predicament still—where is the *causa causarum*?—whence the source of the depressed vital power?—why chase the endless circle?—why urge transcendental arguments, which no one can contradict, but which no one feels to be either satisfactory or elucidatory?

"4th. *Inflammation if it exists is not of a very active nature, and will not bear antiphlogistic treatment.*" As a general enunciation, we here cordially agree with the writer.

Dr. Munro admits that, "*it would be absurd, and apart from the truth, to deny the presence of great congestion of blood in the head in acute mania,*" but regards it rather as the effect than the cause of nervous irritation. In this particular he coincides with Crichton, who wrote in 1798 as follows: "Increased vascularity, diminished vascularity, coloured spots, increased density, increased specific gravity; prætermatural laxity, ulceration, &c., have been detected in various cases, yet there is no one which has been uniformly present in all analogous cases, and therefore, there is no reason to believe, that any one of them is to be considered as the immediate cause of the alienation of mind, but *rather as accidental effects, arising from various causes, which have occurred either previous to the commencement of the disorder, or during its attack.* The chief circumstance, however, which proves that they are rather *consequences* than causes of *any particular disease* is, that they have been found not only in phrenitic patients, but also in idiots, melancholics, hysterical patients, paralytic ones, and epileptic people."—*Crichton on the Nature and Origin of Mental Derangement.* These opinions have been acquiesced in by nearly every writer on mental diseases; and certainly all modern observers fully agree with the able writer before us, in the treatment recommended to be pursued—namely, local depletion accompanied by a careful attention to whatever may support the strength of the patient—regarding the loss of any blood as a great evil—to be avoided whenever possible, and to be modified in every case by judicious hygienic arrangements.

In the arguments derived from the necroscopy of the insane, the author has no new facts to promulge. We were startled, however, at the assertion, that "the congestion and inflammatory action (admitted to exist) are not dangerous to life." Deriving his facts from the records of the incurables of Bethlem Hospital, who have passed through the acute and more dangerous stages of the malady, and who, moreover, never presented the disease in the dire form which it is met with in other hospitals (the patients received at Bethlem being always "picked cases," free from paralysis, epilepsy, and the like), he has given currency to the great error, that "the alterations which take place in insanity are not of a very fatal character." In a foot-note, this error is modified, but the whole paragraph implies, that the *viability* of the insane is at least *equal* to the average of the population. We had thought that the profound researches of Farr, Thurnam, and others, had proved the fallacy of this opinion, and set the question at rest for ever. Dr. Farr, in a table published in the "British Annals of Medicine," has the following:—

"The annual mortality among lunatics was 9 per cent.; the annual mortality of the Swedish population, at the age of 40—45, was 1.50 per cent. It need be scarcely added, that at this age the mortality of the Swedes differs inconsiderably from that of other European nations: *madness, therefore, increases the mortality sixfold.* But it is necessary to show that the mean age of lunatics does not *exceed* 40—45 years: as there are not observations sufficient to determine their mortality at different ages. The mean ages of 977 patients, admitted in 5 years at Bethlem Hospital, were, in 1830, 37 years; in 1831, 35; in 1832, 37; in 1833, 36; in 1834, 36; so that 40—45 may be safely taken as representing the ages of the entire class—those above as well as those below that central point."—(June 16, 1837.)

Dr. Thurnam, in his "Statistics of Insanity," writes: "In those connected with the Society of Friends, less than two-thirds, and, in the others, not more than a third, of the expectation of life, at the time of attack, was, on an average, realized. This is one way in which the prejudicial influence of insanity upon the duration of life may be shown."—p. 101.

Dr. Hitchman, in one of his clinical lectures, delivered at the Hanwell Asylum, and published in this Journal in April, 1850, states: "The mortality in this asylum during the past ten years has been 7·78 per cent.; and I find, from a most valuable article on Lunatic Asylums in the 'Supplement of the Penny Cyclopedia,' that it has, in the Norfolk Asylum, from 1836 to 1845, reached as high as 19·74 per cent.; and at Lancaster, 14·94 per cent.; and Stafford, nearly as high; the mean of 44 asylums in England, Ireland, and Wales, being about 9·62 per cent." . . . . . "A tolerably extensive experience among the insane enables me to state, that, with the exception of fever, there is no disease which they are not as liable to as the general population, while they incur the additional risks of the affections incident to mental derangement; and, therefore, with all these facts before you, you may positively affirm that insanity *has* a tendency *to shorten the duration of human life;* nay, that it increases the mortality at least threefold."—p. 231.

We pass over Dr. Munro's observations on the relation that deteriorated blood may bear to insanity, with the simple remark, that, like the above statistics, in relation to his theory, his arguments are frequently conflicting,—thus, if the insane live the average duration of human life, where is the proof of a low vitality? Contrast the following as an illustration of theory-building. "That external agents act as exciting causes to this state of loss of vigour, but that both the *predisposing* and *proximate cause is in the organ itself.* And (anticipating somewhat what I have to say on the relation which I believe deterioration of blood to hold in the pathology of the insane) I would say, in the terms of microscopic anatomy, that I believe the fault to exist in the nerve-model rather than in the matter assimilated."—Part I., p. 75, with: "For though we have every reason to believe that the nerve-model retains an undeviating power as long as it exists; and that if the circumstances in which it was placed only gave it the opportunity, it would certainly assimilate to itself proper materials, and in the right manner, we cannot believe that it can effect normal tissue, when the proper ingredients are not presented to it; and, consequently, we must believe that, *be the nerve-model ever so perfect*, deteriorated blood must have an effect on its operations."—p. 103.

This mistake is the one, committed by pathologists generally, in reasoning upon insanity; there is no *one special demonstrable* cause in the production of this malady; if "the nerve-model be perfect," and the blood be diseased, surely the "predisposing cause" is in that fluid? If the nerve-model be subject to pressure, is not pressure the "predisposing cause" of its disordered function? No unity is violated—no law of strict induction is infringed by stating, at one time, that an inflamed pia-mater is the cause of insanity, and, at another, that a thickened cranium is the *fons et origo* of the malady, or that the superficies of the brain, like the grey arc of the spinal cord, can be irritated by a reflex action from remote organs, since all of these agents may operate by inducing irritation or derangement in *one* special structure. Until some such views as these are taken by medical men, their theories will be conflicting and inharmonious, and their practice empirical and unsafe. We had much to add on this most interesting topic, but our present space would not enable us to do justice to so great a theme. We cannot, however, conclude our review without noticing another statistical error into which our author has been led, by riding his most excellent hobby a little too fast—"*The female sex being particularly prone to insanity, corroborates the theory.*" This theorem leads to many arguments, but is the premise true? There is a tradition extant, that King Charles puzzled the "big-wigs" of the Royal Society by asking, "how it was that a couple of live fish, placed in a bucket of water, did not make it heavier?" It was a long time before the peerless philosophers detected that the question involved a fallacy. It is a great truth, that "there are more false facts than false theories," and we are strongly inclined to suspect that in the above theorem, a "false fact" is honestly brought forward to support a fallacious theory. "The wards of Bethlem Hospital show, that with equal accommodation for the male as for the female sex, with an equal ease of admission, and with equal means of cure, a far greater number of females are received within these walls than of males." "During the twenty-nine years ending December, 1848, 3,979 females were admitted, while only 2,657 males were received, *i.e.*, *nearly fifty per cent. more females than males.*" It is perfectly astonishing that men should jump to conclusions from such a fact as the above, without first ascertaining what *proportion the female sex may bear, in point of numbers, to the entire sane population of the country.* Of what value are statistics, unless *all* the facts are taken into consideration? In a country like England, where there is always an excess of females, is it not likely that the actual number of insane females should be greater than that of males, without proving that, *comparatively*, they were more liable to insanity than men? What are the facts?

In the census of 1841, the actual excess of females over males in the population of England, was 348,364. The returns of the Commissioners in Lunacy on January 1, 1844, give the proportions of each sex *then in asylums*, as men, 5521, and women, 5751; where is the especial proneness to be found in the above facts? For be it remembered, the *mortality* among the insane males is greater than among females, and if the number of *admissions* into all the asylums during the year had been given, we doubt not but that the actual number of males would have been the larger, without taking the comparative number of each sex among the sane population into consideration. In the Commissioners Report of 1847, there were 7055 males, and 7350 females; bearing then in mind the difference in the sane population of the two sexes, and above all, considering the *great annual mortality* among males, as compared with that of females, what becomes of "the 50 per cent. more females than males;" the great colossal prop of a dazzling and brilliant theory? It is true that the author supports his statistics by reference to the numbers in St. Luke's Hospital, and by the illustrious name of Esquirol. These are *all* his facts on this subject. A great name often misleads. Esquirol's mistake has dragged with it the names of all those who have compiled theses upon a malady with which they were not personally familiar. Copland and Prichard (the latter before he was commissioner) repeated the error of Esquirol, and they have been followed by many others. What were Esquirol's statements? Simply, that the number of *existing cases* of insanity were as thirty-seven males to thirty-eight females; and what is this? admitting that in the general population of the countries from which he derived his data, the proportion of adult females in the general population would probably be greater than that of males, especially after a severe and protracted war. Neither Bethlem, nor St. Luke's would be a good criterion on this subject, for in Middlesex in 1841, there was in the general population an excess of 98,828 fèmales; yet, notwithstanding this disparity of numbers, the returns from the county asylum (Hanwell) show, that during the past twenty years, there have been admitted into that institution 1732 males, and only 1647 females, notwithstanding that there is more ample accommodation for the latter than the former. Such are the conflicting results to be arrived at, by taking a limited sphere, and a brief space of time, for the collection of facts from whence to draw important inferences. We may add, that Earle makes a return from the United

States of 4510 insane men, and 2480 insane women; that Dr. Maximilian Jacobi reported from the Prussian provinces on the Rhine, in 1824, the existence of 1180 males, and only 835 females; and that Dr. Thurnam's laborious inquiries have induced him to conclude, that there is an excess of 13·7 per cent. of males over that of females, in the cases admitted to the various asylums of this kingdom. The researches of M. Bouteville and Parchappe accord, in their general result, with the above; a conclusion which is in accordance with what, *à priori*, might be supposed by a reflecting mind, although not in harmony with the conclusions which a romantic fancy, or a speculative imagination, or a novel theory, might anticipate or require.

We have alluded to these errors with all the more freedom, because the book is no everyday production. It reminds us, both by the graces of its style and the plausibility of its hypothesis, of another great work, the "Indications of Insanity," in which genius and scholarship have combined to weave a theory, difficult indeed to refute, but which, nevertheless, like the one before us, fails to carry conviction to such minds as are daily conversant with the endless phenomena embraced in that comprehensive word, "INSANITY." Like that work, too, the "remarks" are full of sentiments which do honour to the head and the heart of the writer. What can be more beautiful than the following:—

"We cannot listen to Nature's voice too anxiously; we cannot be sufficiently jealous of allowing theoretic science and learned egotisms to carry us out of her track. But if the influence of nature and her gifts be such upon the watching friend, what are its effects and associations upon the sick man himself? The very thought of these things is like a light illuminating the solitude of a dungeon. He who has explored those mysterious solitudes of the earth, the caverns in Derbyshire, may remember, perhaps, a sense of oppression ever increasing, as he descends deeper and deeper into those gloomy regions. The faint light of his conductor would show him that he was, indeed, passing through a dismal solitude; and he might well say, in the language of Scripture, 'I went down to the bottom of the mountains; the earth with her bars was about me for ever!' The rush and hollow sound of waters as they fell around him into deeper caverns still, might well occasion him to feel that chaos surrounded him, and that he was cast out, and forsaken; when suddenly, as if by magic, a crown of lights is raised up into the solitudes above! All is changed in a moment. The eye turns with instinctive fondness to those glowing stars; what appeared the chamber of death is changed into a glittering room; the terrific fall of waters becomes a beautiful cascade; chaos seems to have departed, and hope returns. Such as these beautiful lights are to the adventurer, the associations, and the *effects* of nature's gifts are upon the sick, and ill at ease; indeed, far more; for no temporary gloom can equal the shadow cast upon the mind of him whose nervous system is distressed, and nothing can seem so bright as the associations and sensations of returning health.

"And let me add, in conclusion, a reflection which the circumstance that these remarks refer to the distresses of the mind peculiarly justifies, and which the analogy just given almost forces upon the attention, namely, this, that if the traveller—his journey through earth's solitary places, his joy when the light breaks in upon his gloom, his sadness when he perceives that light expiring—remind us of man's sojourn upon earth—the trials of his life, the solace permitted in the right use of nature's gifts, and the regrets experienced when these comforts are taken away;—if, I say, these vicissitudes of the adventurer remind us of the changing scenes of life's fevered dream, and a sense of cheerlessness is left upon the mind, this most happy thought remains, that as the traveller can cast away all the gloom of the cavern, its pleasures and regrets, by the knowledge that they are but temporary, and that by retracing the dim and rugged path by which he descended, he can regain the portals that open to life and home once more; so the man of devout mind can derive lasting consolation from the thought, that when life's journey is over, when the worst and the best have been tried and found wanting, an entrance is granted to him into a home more blessed and enduring far, than this world can ever offer."—p. 144.

Our space will not permit us to follow the author through any more of his fascinating pages; we had marked many passages for approbation, and should have been glad to have placed before our readers some more extracts of a kindred spirit to those which adorn the first and concluding portions of this review, but are unable to do so with justice to other writers, who are at present claiming our attention. We trust, however, that each reader will purchase the original work; it possesses the great merit of being of small size, and abounds with deep thoughts eloquently expressed, and so richly imbued with a Christian spirit, that they cannot fail to make every careful reader a wiser and a better man. With its pathology we cannot agree, but the style and the spirit of the volume merit and possess our warmest approbation, and we trust again to meet the author, when after years have matured his experience, and his fervid imagination has become more submissive to the stern requirements of the judgment, and to the rigid laws of an inductive philosophy.

